# Methamphetamine intoxication in a dog: case report

**DOI:** 10.1186/1746-6148-10-139

**Published:** 2014-06-24

**Authors:** Zengyang Pei, Xu Zhang

**Affiliations:** 1 Department of Veterinary Medicine (Small Animal Section), College of Animal Sciences, Zhejiang University, 866 Yuhangtang Road, Hangzhou 310058, PR China; 2 Ai’ xin Small Animal Hospital, 180-182 Jiaochang Hills Road, Cixi 315300, PR China

**Keywords:** Methamphetamine, Intoxication, Dog, Agitation, Tachycardia, Hyperthermia, Disseminated intravascular coagulation, Urine screen test, Gas chromatography mass spectrometry

## Abstract

**Background:**

Methamphetamine abuse has undergone a dramatic worldwide increase, and represents a significant and global issue for public health. Incidents of methamphetamine intoxication and death in humans are relatively commonplace. Because of its increasing illicit availability, together with legitimate use in human medicine, accidental or intentional exposure to methamphetamine in dogs is becoming a more likely scenario.

**Case presentation:**

A 3-year-old, 3.7 kg intact female Miniature Poodle who had been intentionally fed an unknown amount of a crystalline-like substance developed extreme agitation, seizures, tachycardia, hyperthermia, hypertension, disseminated intravascular coagulation (DIC), bloody diarrhea, and dilated pupils. Blood work revealed leukocytosis, erythropenia, lymphocytosis, thrombocytopenia, coagulation abnormalities, but all to a mild extent, together with mild elevation in both alanine aminotranferease (ALT) and alkaline phosphatase (ALKP), and a mild decreased in glucose. Radiologic diagnosis revealed generalized, severe distension of the stomach and small intestinal tract with air. Immunochromatographic screening tests and gas chromatography mass spectrometry analysis confirmed methamphetamine intoxication and revealed concentrations of methamphetamine in blood and urine of 0.32 μg/mL and 2.35 μg/mL respectively. The dog demonstrated progressive improvement after supportive care, with the high fever resolved over the initial 24 hours of hospitalization, and agitation was successfully controlled beyond 48 hours after initial hospitalization. Hemostatic abnormalities were progressive improved after heparin therapy and supportive care. By the sixth day of hospitalization the dog was clinically well, and all laboratory data had returned to normal with the exception of a mild elevateion of ALKP.

**Conclusion:**

To the authors’ knowledge, this is the second case report of methamphetamine intoxication in dogs presented in veterinary practice in open literature so far. Although rare, methamphetamine intoxication should be considered as a differential diagnosis in dogs with a toxic substance ingestion history and with typical nervous and cardiovascular system symptoms. In such cases rapid diagnosis and aggressive intervention is important for prognosis. Blood methamphetamine concentration may be a helpful value for assessment of the severity of intoxication and prediction of clinical outcomes.

## Background

Methamphetamine, a potent central nervous system (CNS) stimulant, was first synthesized around 1920 [1] and widely used in the 1930s for the medical treatment of depression, obesity, sleep disorders, nasal congestion, and many other conditions in humans [2-4]. An increasing awareness of the adverse health effect associated with methamphetamine led to the withdrawal of many previously sanctioned uses of the drug and the addition of the prohibitions relating to unauthorized possession, use or distribution of the drug in many countries. Nevertheless, methamphetamine abuse has been rising over the past decades and has become a serious worldwide health issue. This is considered to be due to its ease of synthesis, requiring only rudimentary and affordable laboratory equipment and readily available ingredients for its production, and its resulting highly addictive properties [5-7]. Currently, methamphetamine is one of the most widely used illicit drugs in China, and its use seems to be increasing [8]. Moreover, medicinal methamphetamines still remain in limited use for the treatment of narcolepsy, attention deficit hyperactivity disorder (ADHD), and obesity [4,9,10]. This escalating availability and use in humans together with the fact that there are probably more than 200 million dogs in China [11], may lead to the increasing likelihood of accidental or intentional methamphetamine exposure in dogs.

It is now well reported in humans and several other animal models that methamphetamine intoxication is associated with a number of adverse consequences including agitation, aggression, hyperthermia, delirium, cardiac ischemia, seizures, strokes, rhabdomyolysis, dysfunction of the liver, heart, kidney, or even death by overdose [12-17]. So far, however, incidences of methamphetamine intoxication in dogs seem to have had relatively poorly covered, with only one intoxication case report presented in 1998 [18], two toxicity studies reported in 1965 [19] and 1982 [20], three pharmacokinetic studies conducted from 1995 to 1997 [21-23]. Beyond these there remain very few case reports relating to primary metabolite amphetamine intoxication [24-26] published and available in English. Herein, we report an unusual methamphetamine toxicity case in a Miniature Poodle dog that had ingested unknown amounts of methamphetamine.

## Case presentation

A 3-year-old, 3.7 kg intact female Miniature Poodle was taken to the Ai’ xin Small Animal Hospital (Cixi, Zhejiang, China), with a heart rate of 138, respiratory rate of 62, rectal temperature of 42°C, and intermittent seizures. The owners initially presented a conflicting and unclear history. The dog received external cooling without any other examination or treatment after admission. After continued conversation with the owners it became clear that the dog may have been intentionally fed an unknown amount of a crystalline-like substance by their friends in a nightclub approximately 1 hour before presentation. They suggested that ‘ice’, the street name of methamphetamine, may have been present in the nightclub. The dog had no medical records in the hospital, but, according to the owner’s description, had no other remarkable past history. After revealing a possible methamphetamine ingestion history the owners disappeared, leaving the dog at the hospital.

**Figure 1:**
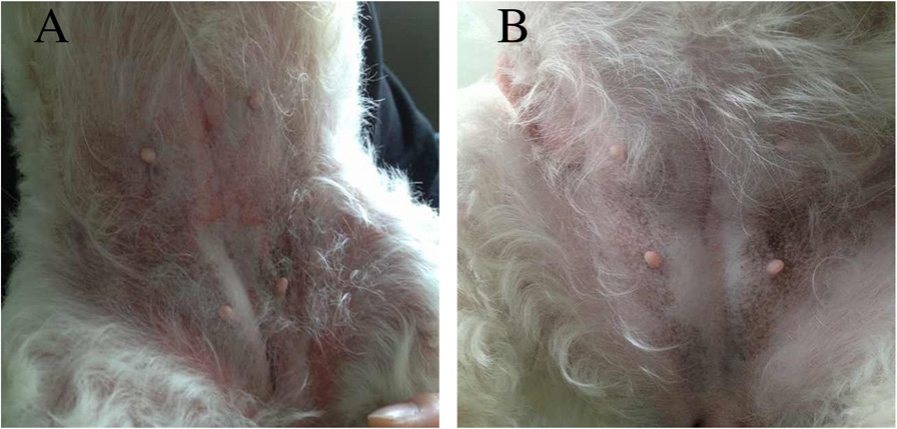
**Skin flush of a dog after unknown amount of methamphetamine ingestion.** The dog progressive presented with skin flushing and petechiae after ingestion **(A)**, and become normal after treatment **(B)**.

The dog was taken to the Veterinary Teaching Hospital of Zhejiang University by a referral veterinarian, and was provided with continuing external cooling (ice packs). By this time it was about 2.5 hours beyond ingestion when the dog was finally presented at the emergency room. The referral veterinarian reported that the dog was progressively displaying flushed skin and extensive petechiae in abdominal region (Figure 1). It also had started to develop watery bloody diarrhea, and had had 3 short seizures (about 50 seconds for each time seizure) on the way to the hospital. Physical examination revealed a rectal temperature of 41.5°C, heart rate of 186 beats per minute with no premature ventricular contractions or other arrhythmias, panting respiration, systolic blood pressure of 166 mmHg, diastolic blood pressure of 117 mmHg, and mean arterial pressure of 137 mmHg. The dog was alert, responsive and extremely agitated. It had bilaterally dilated pupils and displayed rotational locomoter activity (repeatedly circling in an anticlockwise direction) (Additional file 1: Movie 1).

Blood samples were collected by cephalic venipuncture and placed in different sets of tubes, with or without anticoagulant, for a complete blood count (CBC), a biochemical profile panel and coagulation tests. All these tests were performed by IDEXX In-house Diagnostics Station (IDEXX Laboratories, Sacramento, CA). Hematological findings included white blood cell count 21,000/μL (reference range 5,050-16,760/μL), red blood cell count 5.1 M/μL (reference range 5.65-8.87 M/μL), red blood cell specific volume 31.2% (reference range 37.3%-61.7%), platelets 55,000/μL (reference range 148,000-484,000/μL), lymphocytes 5,300/μL (reference range 1,050-5,100/μL), and neutrophils 15,000 (reference range 2,950-11,640/μL) with normal segmented neutrophils. The results of the biochemical panel revealed mildly elevated alanine aminotranferease (ALT; 112 U/L, reference range is 10-100 U/L) and mildly elevated alkaline phosphatase (ALKP; 238 U/L, reference range is 23-212U/L) concentrations, with mildly decreased glucose (70 mg/dL, reference range 74-143 mg/dL). The prothrombin time (PT) was 16.8 seconds (reference range 11.0-14.0 seconds) and the activated partial thromboplastin time (aPTT) was 125.0 seconds (reference range 60.0-93.0).

**Figure 2:**
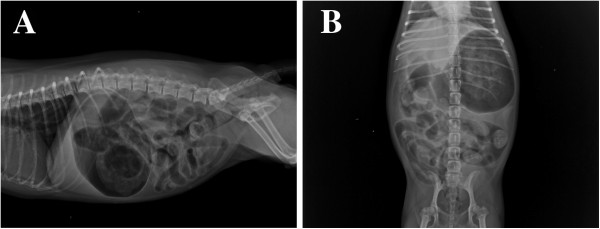
**Radiograph of the abdominal region in a dog with methamphetamine intoxication. A**: Right lateral view. **B**: Ventral dorsal view. Note generalized, severe distension of the stomach and small intestinal tract with air. Portions of the colon were also air filled. Presumptive diagnosis was functional or paralytic ileus.

**Figure 3:**
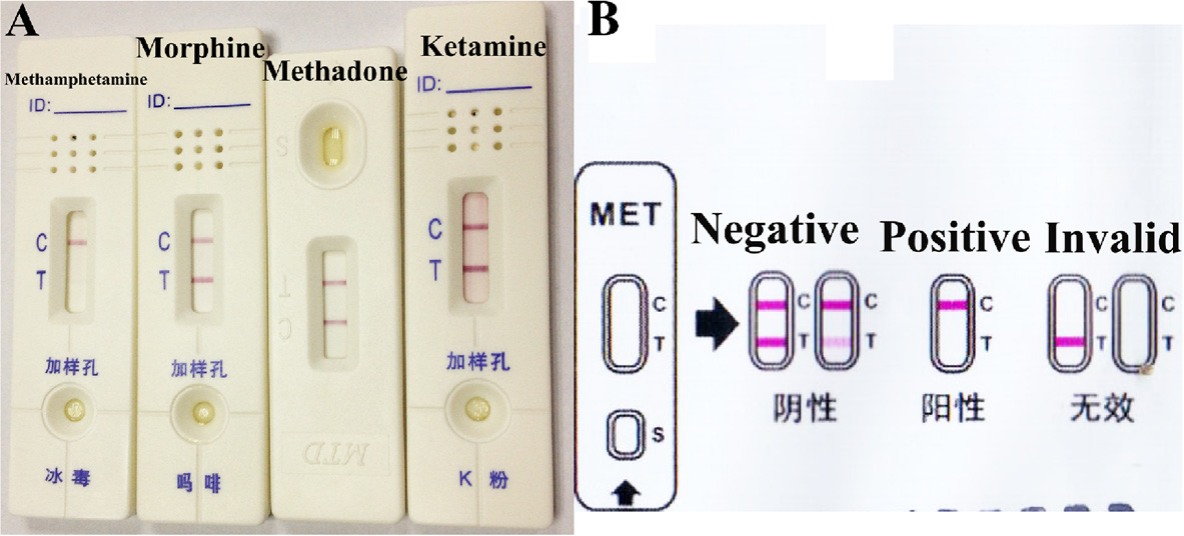
**Rapid competitive immunochromatographic screening tests for the detection of ketamine, methamphetamine, methadone, or morphine.** The screening tests showed positive results only for methamphetamine **(A)**. The interpretation of test **(B)** is based on the pink coloured bands appearing at the control region (C) and at the test region (T). Two pink coloured bands appearing at C and T region indicates absence of methamphetamine in the specimen. Only one pink coloured band appearing at the C region indicates the specimen contains a detectable amount of methamphetamine. If no band appears at the C region, this indicates the test result is invalid.

Blood gas was also evaluated by electrolyte analysis and a blood gas analyzer (IDEXX Laboratories, Sacramento, CA) and the results revealed a pH of 7.20 (reference range 7.31-7.42) and a base deficit of 17.8 mmol/L (HCO_3_^-^, reference range 20.0-29.0 mmol/L), with unremarkable potassium, chloride and sodium concentrations. An x-ray of the chest and abdomen appeared normal with exception of severe distension of the stomach and small intestinal tract with air. Portions of the colon were also air filled. Presumptive diagnosis was functional or paralytic ileus (Figure 2). The urine test was unremarkable without any pathologic casts.At the time of presentation at the emergency unit, a preliminary drug screen on urine was performed using rapid competitive immunochromatographic screening tests (ABON Biopharm, Zhejiang, China) for the detection of ketamine, methamphetamine, methadone, or morphine. The screening tests showed positive results only for methamphetamine (Figure 3A). At the same time, the blood and urine samples were collected and submitted to Zhejiang Forensic Medical Examination Center (Hangzhou, Zhejiang, China) for the confirmation and quantification of the methamphetamine present in blood using gas chromatography mass spectrometry (GC-MS). The GC-MS results were provided by the police reports. Quantitative test revealed that the methamphetamine concentrations in serum and urine were 0.32 μg/mL and 2.35 μg/mL, respectively.

After the blood samples were taken, the dog was placed in a quiet room with minimum external stimulation. In order to treat the hyperthermia and maintain an adequate urine output, a venous catheter (Introcan; B. Braun Trading Corp, Shanghai, China) was placed in one cephalic vein for fluid administration, using 0.9% Sodium Chloride Solution. External cooling was continued using ice packs, and the rectal temperature was continually monitored to prevent possible hypothermia. The dog was given 3.6 mEq of intravenous sodium bicarbonate to correct the base deficit over the first 12 hours. The dosage then was adjusted according to the subsequent blood gas analysis. An intermittent intravenous injection of 50% Glucose Solution was given as appropriate based on continuous monitoring of serum glucose for the regulation of mild hypoglycemia. Subcutaneous amoxicillin and clavulanate potassium (7 mg/kg amoxicillin and 1.75 mg/kg clavulanate potassium as effective; Synulox Injection, Pfizer, New York) was begun on admission, and withdrawn within 4 days after the white blood cell count returned to normal and after confirming that urine cultures proved no growth of bacteria. Intravenous phenobarbital (4 mg/kg; Sodium Phenobarbital Injection, Tianjin Pharma., Tianjin, China) and acepromazine (0.05 mg/kg; Acepromazine injection, Mavlab Pty Ltd, Queensland, Australia) were given to control seizures and agitation. The dog was started on low-molecular-weight heparin to control coagulation abnormalities, supported with intravenous fluids for maintaining sufficient microvascular perfusion.

Over the initial 24 hours of hospitalization, the dog demonstrated progressive improvement, with the high fever resolved (between 36.5 and 37.6°C), normal serum glucose concentration, and sufficient urine output. After administration of phenobarbital and acepromazine the dog was lay down in the cage and ceased its repetitive circling. Nevertheless, it still had several short seizures and displayed occasional muscle twitching. Following the repetitive administration of phenobarbital and acepromazine no more seizures, muscle twitching, or repetitive circling behaviors were observed (beyond 48 hours after hospitalization). Urine was screened again on day 3 using the same rapid assay and at that time failed to reveal the presence of methamphetamine. By the sixth day of hospitalization the dog was clinically well (no diarrhea, no petechiae, no pupillary dilation, and eating and drinking well) and all laboratory data had returned to normal with the exception of a mildly elevated ALKP (220 U/L, reference range 23-212 U/L). The dog was sent to local veterinary hospital with no further medications.

## Discussion

As methamphetamine abuse continues to rise due to its increasing availability, the likelihood of accidental poisoning in companion animals increases. Therefore, a better understanding of methamphetamine intoxication for small animal veterinary practice is urgently needed. Methamphetamine toxicity predominantly affects the nervous and cardiovascular systems [3,4,12]. Following oral administration, methamphetamine is well-absorbed into the bloodstream, metabolized in the liver, and excreted by the kidney both as methamphetamine in its unchanged form and as its main metabolite amphetamine. Both methamphetamine and amphetamine directly or indirectly trigger the release of dopamine from neurons in the CNS. This leads to stereotypical behaviors such as rearing, licking, and gnawing, and other forms of increased locomotor activity in animal models [4,12,27]. Similar to the neurotoxic effects on the dopamine system, methamphetamine can also affect glutamatergic, serotonergic and noradrenergic neurotransmission, which subsequently contributes to stimulation of the sympathetic nervous system with tachycardia, hyperthermia, hypertension, agitation, and dilated pupils resulting [3,4,12]. In this case, the most prominent presenting symptoms were agitation, with signs of restless, short seizures, and rotational locomoter activity (circling). The dog also presented bilaterally dilated pupils, hypertension, hyperthermia, and tachycardia. All these symptoms were consistent with previous studies of methamphetamine toxicity in dogs [18-23].

Moreover, the skin petechiae of the dog, together with the mild thrombocytopenia, prolonged PT and aPTT, were consistent with disseminated intravascular coagulation (DIC). DIC was reported to be found in methamphetamine or amphetamine toxicity in both dogs and humans [3,25,27]. The mechanism of methamphetamine-mediated DIC is unknown but it is probably associated with hyperthermia because previous studies have shown that thrombocytopenia and coagulation abnormalities are commonly seen in dogs with fever and heat-induced illnesses [28-30]. Methamphetamine has also been associated with severe abdominal pain, vomiting, bloody diarrhea, and gut ischemia in humans, especially when it was taken orally [7,12]. Recently, unusual cases of methamphetamine-associated intestinal infarction, paralytic ileus, and ischemic colitis have been reported in human patients [31-33]. The mechanism by which methamphetamine can induce gastrointestinal disorders has been proposed to be by direct affects on cocaine-regulation and upon the amphetamine-regulated transcript peptide as well as indirect effects from the release of dopamine and other neurotransmitters [34,35]. Similarly, our case presented with frequent bloody diarrhea and severe distension of the stomach and small intestinal tract with air during the first 2 days of hospitalization. Portions of the colon were also air filled. Further examinations such as computed tomography scan, mesenteric angiography, colonoscopy, or tissue biopsy may be options for diagnosis of acute intestinal ischemia, intestinal infarction and paralytic ileus. In this case, we did not perform these further examinations because of the low budget and also because the dog improved quickly with supportive care. Although we were not able to identify the definitive pathogeny of the gastrointestinal disorder in this dog, the presumptive diagnosis of functional or paralytic ileus can be made given the previous reports [31-33].

The diagnosis of methamphetamine toxicosis should be based primarily on the recognition of the clinical symptoms of extremely agitation, seizures, tremors, persistent tachycardia, hypertension, hyperthermia, and other sympathominetic effects [3,18,24,26]. Other drugs such as cocaine, ephedrine, pseudoephedrine, methylxanthines, or strychnine can cause similar CNS stimulation must be included in a differential diagnosis list for methamphetamine toxicosis. Details of ingestion history within 0.5-3 hours before the first episode of symptom can be very helpful for diagnosis, as ingestion is the primary route for methamphetamine administration in companion animals. Ingested methamphetamine may take about 20 minutes to induce symptoms with the peak plasma methamphetamine concentrations achieved at approximately 2-3 hours post ingestion [27,36]. Other supporting laboratory tests such as CBC, blood gas, electrolytes, electrocardiogram, serum glucose, biochemical panel, and coagulation status should be performed as clinically indicated.

When taken orally, 30-54% of the dose is excreted in urine as methamphetamine and 10-23% as amphetamine [36]. Therefore, urine qualitative testing for methamphetamine or its metabolite amphetamine may confirm methamphetamine intoxication and possibly exclude other common illicit drugs as factors. The drug screening kits used in this case are rapid, qualitative, immunochromatographic assays for the detection of the presence of illicit drugs in urine specimens, at concentrations as low as 1000 ng/mL for methamphetamine and ketamine, or 300 ng/mL for methadone and morphine. These kits are based on the principle of agglutinating sera on membrane and utilize the technique of competitive immunochromatography, as previously described [37]. The test procedure takes around 10 minutes and was performed in several simple steps. A negative result produces two pink coloured bands, one at the control region (C) and the other at the test region (T). In contrast, the absence of this pink coloured band in the T region indicates the specimen contains a detectable amount of methamphetamine. For the test to be valid, the C region must show a pink coloured band (Figure 3B).

Although the validations of these urine screening tests for use in companion animals have not been confirmed, this may be because the incidences of illicit drug toxicosis in companion animals are relatively rare and it may be considered not worth the effort to confirm the validation. However, one study in 2009 confirmed that the screening test kit did successfully identify methamphetamine/amphetamine, barbiturates, opiates, and benzodiazepines in urine from dogs that had received these common illicit drugs either intravenously and/or orally [38]. In our case, the screening tests showed positive results only for methamphetamine consistent with the ingestion history and clinical symptoms. Moreover, the blood and urine samples of the dog were collected and submitted for quantitative GC-MS analysis, which allowed positive structural identification and quantitation of the analyte that produced the positive result in the screening tests. The concentration of methamphetamine was 0.32 μg/mL in blood, and 2.35 μg/mL in urine. The results of screening tests and GC-MS analysis were strongly suggested methamphetamine poisoning in the present case.

There is no specific antidote for methamphetamine intoxication and treatment is generally supportive care [3,18,24,26]. Gastrointestinal decontamination with activated charcoal is an early therapeutic option if the animal is seen within 30 minutes of ingestion. Blood pressure, respiration, heart rate and rhythm must all be strictly monitored and treated as appropriate. Hyperthermia must be diagnosed rapidly and treated aggressively using intravenous fluids, fans, ice packs, or cool-water baths, as severe persistent hyperthermia is associated with poor clinical outcome [24,27]. Agitation or seizures should be treated initially with benzodiazepines, or phenobarbital and/or propofol if seizure activity persists. Acepromazine has also been recommended in such cases with dosage titrated to achieve adequate sedation [24]. Extreme agitation and frequent episodes of seizures have also been associated with poor outcomes, even suddenly death, post ingestion [15,18,27]. If the animal presented with myoglobinuria and other signs of rhabdomyolysis, aggressive crystalloid fluid must be used appropriately to maintain renal function, assure adequate urinary output, and promote the elimination of methamphetamine and its metabolites. Gastrointestinal decontamination was not applied in this case because it had been about 2.5 hours post ingestion before the dog had been presented at our emergency room, and the dog presented prominent clinical signs. The dog demonstrated progressive improvement after supportive care, with the high fever resolved over the initial 24 hours of hospitalization, and agitation successfully controlled beyond 48 hours of hospitalization. By the sixth day of hospitalization the dog was clinically well, and all laboratory data had returned to normal with the exception of mildly elevated of ALKP.

The prognosis of methamphetamine intoxication generally depends on the severity of poisoning. Toxic doses of methamphetamine vary widely in different species, with the median lethal dose (LD_50_) reported as 70 mg/kg (via intra-peritoneal injection) for rats, 43 mg/kg (intra-peritoneal injection) for mice. 140-1650 mg (oral or intravenous injection) is considered potentially lethal for humans [39]. According to a previous study, the oral LD_50_ for methamphetamine hydrochloride in dogs can be range anywhere from 9 to 11 mg/kg [19]. The concentration of methamphetamine in the blood is often determined to assess the severity of methamphetamine poisoning. There are several human medical studies which provide a three-fold classification of methamphetamine concentration in the blood into fatal, toxic and therapeutic concentrations [40,41]. Although the results of those studies were not perfectly matched, the therapeutic methamphetamine blood concentrations were estimated to range from 0.01 to 0.05 μg/mL, the toxic concentrations from 0.6 to 5.0 μg/mL, and the lethal concentrations as above 10 μg/mL. In this case, the methamphetamine concentration in blood reached 0.32 μg/mL, which exceeds the therapeutic concentration of this classification despite the dog recovery uneventfully. This suggested that the therapeutic blood concentrations for methamphetamine in humans and dogs may differ, although no studies have been done yet to test either therapeutic or toxic methamphetamine blood concentrations in dogs.

## Conclusion

In conclusion, our case report describes a dog that had ingested an unknown amount of methamphetamine. This led to a syndrome characterized by extremely agitation, seizures, tachycardia, hyperthermia, hypertension, DIC, bloody diarrhea, and dilated pupils. Urine screening tests and GC-MS analysis confirmed methamphetamine intoxication in this dog, with the concentrations of methamphetamine in blood and urine of 0.32 μg/mL and 2.35 μg/mL respectively. To the authors’ knowledge, this is the second case report of methamphetamine intoxication in dogs presented in a veterinary practice context in open English literature so far. Although rare, methamphetamine intoxication should be considered as a differential diagnosis in dogs which have toxic substance ingestion history and that present with typical nervous and cardiovascular systems symptoms. Here, rapid diagnosis and aggressive intervention is important for prognosis. Blood methamphetamine concentrations may be helpful for assessment of the severity of intoxication and the prediction of clinical outcomes. Further studies need to be done to classify the blood methamphetamine concentration ranges for fatal, toxic and therapeutic concentrations in dogs.

### Consent

Consent was obtained from the present owner of the dog for publication of this case report and any accompanying images.

## Abbreviations

CNS: Central nervous system; ADHD: Attention deficit hyperactivity disorder; CBC: Complete blood count; ALT: Alanine aminotranferease; ALKP: Alkaline phosphatase; PT: Prothrombin time; aPTT: Activated partial thromboplastin time; GC-MS: Gas chromatography mass spectrometry; DIC: Disseminated intravascular coagulation; LD_50_: Median lethal dose.

## Competing interests

The authors declare that they have no competing interests.

## Authors’ contributions

ZP performed the clinical examination, was responsible for the hematological diagnosis and urine screening tests, supervised the treatment, reviewed the literature and prepared the manuscript. XZ was responsible for the radiologic diagnosis, monitored the temperature, heart rate and blood pressure of the dog, and was involved in ICU supportive care and manuscript revision. Both authors have read and approved the final manuscript.

## Supplementary Material

Additional file 1: Movie 1 Observation of the rotational locomoter activity (repeated anticlockwise circling) post methamphetamine ingestion in a dog. Click here for file
